# Incidence of postpartum haemorrhage defined by quantitative blood loss measurement: a national cohort

**DOI:** 10.1186/s12884-020-02971-3

**Published:** 2020-05-06

**Authors:** Sarah F. Bell, Adam Watkins, Miriam John, Elinore Macgillivray, Thomas L. Kitchen, Donna James, Cerys Scarr, Christopher M. Bailey, Kevin P. Kelly, Kathryn James, Jenna L. Stevens, Tracey Edey, Rachel E. Collis, Peter W. Collins

**Affiliations:** 1 Department of Anaesthetics, Intensive Care and Pain Medicine, Cardiff and Vale University Health Board, Cardiff, UK; 2 1000 Lives Improvement, Public Health Wales, Tyndall Street, Cardiff, UK; 3 Department of Emergency Medicine, Aneurin Bevan University Health Board, Newport, UK; 4 Department of Obstetrics and Gynaecology, Cardiff and Vale University Health Board, Cardiff, UK; 5 Department of Anaesthetics, Intensive Care and Pain Medicine, Betsi Cadwaladr University Health Board, Ysbyty Gwynedd, Bangor, UK; 6 Department of Anaesthetics, Intensive Care and Pain Medicine, Betsi Cadwaladr University Health Board, Glan Clwyd Hospital, Bodelwyddan, UK; 7 Department of Anaesthetics, Aneurin Bevan University Health Board, Newport, UK; 8 Department of Obstetrics and Gynaecology, Abertawe Bro Morgannwg University Health Board, Swansea, UK; 9 Institute of Infection and Immunity, School of Medicine, Cardiff University, Cardiff, UK; 10 Departmentt of Haematology, University Hospital of Wales Heath Park, Cardiff, UK

**Keywords:** Epidemiology, Haemorrhage, Incidence, Measured blood loss, Postpartum

## Abstract

**Background:**

Visual estimation of blood loss following delivery often under-reports actual bleed volume. To improve accuracy, quantitative blood loss measurement was introduced for all births in the 12 hospitals providing maternity care in Wales. This intervention was incorporated into a quality improvement programme (Obstetric Bleeding Strategy for Wales, OBS Cymru). We report the incidence of postpartum haemorrhage in Wales over a 1-year period using quantitative measurement.

**Methods:**

This prospective, consecutive cohort included all 31,341 women giving birth in Wales in 2017. Standardised training was cascaded to maternity staff in all 12 hospitals in Wales. The training comprised mock-scenarios, a video and team drills. Uptake of quantitative blood loss measurement was audited at each centre. Data on postpartum haemorrhage of > 1000 mL were collected and analysed according to mode of delivery. Data on blood loss for all maternities was from the NHS Wales Informatics Service.

**Results:**

Biannual audit data demonstrated an increase in quantitative measurement from 52.1 to 87.8% (*P* < 0.001). The incidence (95% confidence intervals, CI) of postpartum haemorrhage of > 1000 mL, > 1500 mL and > 2000 mL was 8.6% (8.3 to 8.9), 3.3% (3.1 to 3.5) and 1.3% (1.2 to 1.4), respectively compared to 5%, 2% and 0.8% in the year before OBS Cymru. The incidence (95% CI) of bleeds of > 1000 mL was similar across the 12 hospitals despite widely varied size, staffing levels and case mix, median (25th to 75th centile) 8.6% (7.8–9.6). The incidence of PPH varied with mode of delivery and was mean (95% CI) 4.9% (4.6–5.2) for unassisted vaginal deliveries, 18.4 (17.1–19.8) for instrumental vaginal deliveries, 8.5 (7.7–9.4) for elective caesarean section and 19.8 (18.6–21.0) for non-elective caesarean sections.

**Conclusions:**

Quantitative measurement of blood loss is feasible in all hospitals providing maternity care and is associated with detection of higher rates of postpartum haemorrhage. These results have implications for the definition of abnormal blood loss after childbirth and for management and research of postpartum haemorrhage.

## Background

In the UK, postpartum haemorrhage (PPH) is defined by The Royal College of Obstetrics and Gynaecology (RCOG) as minor for bleeds between 500 and 1000 mL, moderate > 1000–2000 mL and severe > 2000 mL [1, 2]. Other groups define PPH > 1000 mL as severe [3–6] and a core outcome set, based on an expert consensus and Delphi analysis, recommended the use of ≥1000 mL as a key outcome measure for treatment and prevention of PPH [7]. Current PPH management guidelines advocate escalation of care to more senior staff based on specific volumes of blood loss, rate of bleeding and patient’s vital signs [1, 8, 9]. In order to apply these guidelines in routine clinical practice, clinicians need to be able to measure blood loss accurately and easily.

Defining the incidence of PPH, and comparing results of studies, is hampered by the lack of a standardised approach to blood loss assessment [2]. Visual estimation, although widely used, is inaccurate and often associated with under-reporting of actual blood loss, especially in cases of large volumes of PPH [3–5, 10–19]. Systematic reviews confirm that the incidence of PPH is higher with quantitative measurement, as opposed to visual estimation and this leads to inaccurate reporting of the incidence and severity of PPH [3–5].

Quantitative blood loss (QBL) measurement, using gravimetric and volumetric techniques, is more accurate than visual estimation, both in simulations and clinical practice [10, 13, 15, 19, 20] and so gives a more representative and reproducible measure of the incidence and severity of PPH, although all methods have shortcomings. It remains unclear, however, whether quantitative measurement is feasible in all clinical settings, after all types of deliveries and outside of routine hours. Furthermore quantitative measurement is not routinely practiced in all hospitals and for all maternities [21].

We report on the incidence of PPH after the introduction of standardised, QBL measurement after all modes of delivery and in all maternity settings in Wales as part of a national quality improvement programme called The Obstetric Bleeding Strategy Wales (OBS Cymru) [22].

## Methods

The lead research and development office (Cardiff and Vale University Health Board) categorised the project as a quality improvement initiative, which did not require ethical approval or individual patient consent to collect and report data. The project was registered within each health care region of Wales as a quality improvement programme and data sharing agreements enabled information to be collected in a national database. Patient and public representatives were members of the OBS Cymru steering committee and were involved in the design and conduct of the quality improvement programme.

Between January and March 2017 the quality improvement programme introduced a package of interventions into all 12 hospitals providing maternity care in Wales. These hospitals provide care in both midwifery- and obstetrician-led settings. A key intervention was the introduction of cumulative, QBL measurement for all births, irrespective of the perceived risk of bleeding. QBL measurement was started at delivery although, in cases of antepartum haemorrhage, bleeding within 24 h before birth was included in the cumulative calculation. QBL measurement was defined as the combined blood loss from all gravimetric and volumetric sources and was continued until the treating clinicians were confident that abnormal bleeding had stopped. The gravimetric technique included weighing all blood soaked swabs and under-buttocks incontinence bed pads and subtracting their known dry weight and has been previously described [8, 15, 20]. At unassisted vaginal delivery, under-buttocks incontinence pads were changed immediately after delivery to discard amniotic fluid, and subsequent fluid on new pads was assumed to be blood and therefore measured. Blood on bed linen was weighed and the dry weight subtracted. For instrumental vaginal deliveries, conical under buttock drapes were placed after amniotomy. Conical under buttock drapes were not used for unassisted vaginal deliveries. In the operating theatre the volume of blood loss collected in suction containers was calculated following consensus agreement regarding the subtraction of amniotic fluid. A national training package was developed to support implementation based on knowledge gained from previous PPH studies at one hospital where accurate measurement was required to define research study entry [23–26]. This included an on-line training package, https://www.youtube.com/watch?v=3aKse0HbAac (accessed 14th Mar 2020) developed in collaboration with the Association of Women's Health, Obstetric and Neonatal Nurses (AWHONN).

At each of the 12 hospitals providing maternity care the local leadership team, consisting of a senior midwife, obstetrician and anaesthetist, designated a midwife with an interest in quality improvement and PPH to be their local champion midwife. The national OBS Cymru senior midwives and medical clinical leadership fellows delivered standardised training to the local midwifery champions. This consisted of scenario based teaching using mock blood loss simulation, teaching video and training drills. The local champion midwives cascaded training to midwives, maternity support workers and theatre teams. OBS Cymru senior midwives delivered training to undergraduate midwives within their university courses throughout Wales.

The accuracy of the method for blood loss measurement was assessed in Cardiff. In 372 maternities, where the mean (range) blood loss was 643 (10–3000) mL, the correlation coefficient between quantitatively measured blood loss and fall in haemoglobin adjusted for red blood cell transfusion was *r* = − 0.57. The correlation was similar in the operating theatre *r* = − 0.58 and the birthing room (including both midwifery and obstetrician-led care) *r* = − 0.55 (manuscript accepted).

Audit cycles to assess uptake of QBL measurement were performed by the 12 hospitals providing maternity care in Wales. Champion midwives reported the method of blood loss measurement for all maternities during a week or for at least 30 consecutive births. The mode and place of delivery, volume of blood loss and method of measurement for each item were recorded. When all items used to collect blood during the delivery were appropriately measured, blood loss was considered to have been quantitatively measured. A baseline audit was performed in October 2016, before OBS Cymru had started, with repeat cycles in June 2017 and December 2017.

Information on blood loss for all maternities in Wales in the year before the introduction of quantitative blood loss measurement and the establishment of the OBS Cymru database was accessed through The NHS Wales Informatics Service (1st April 2015 to 31st March 2016). This anonymous dataset did not link blood loss with mode of birth or hospital site and was automatically complied from local maternity datasets. A national OBS Cymru database was established by 1000 Lives Improvement, the national quality improvement service for NHS Wales, to capture information about maternities where blood loss was 1000 mL or more. Data in the OBS Cymru database included mode of delivery, aetiology of bleeding, whether blood loss was measured quantitatively or estimated visually and total volume of blood loss. The OBS Cymru database did not collect information for PPH < 1000 mL. Information on the mode of delivery for all maternities in 2017 was obtained from the Welsh Maternity Indicators data set [27]. In four maternity units data were collected that linked the volume of blood loss to mode of delivery after all births (as opposed to only for bleeds > 1000 mL) after quantitative measurement had been adopted, these data included both midwifery- and obstetric-led births. The 4 units were the smallest, largest and two medium size maternity units so that a wide spread of practice and case mix could be observed.

Continuous data are analysed descriptively and presented as median, interquartile range and range, mean and 95% confidence intervals (CI). Categorical values are presented as number and percent. The incidence of PPH > 1000, > 1500 and > 2000 mL were calculated using binomial confidence intervals using the Hmisc R library 4.1.1 implementation of the Wilson method. R 3.2 was used for all analyses. Differences in the uptake of blood loss measurement were investigated using Chi square test.

## Results

Between 1st January and 31st December 2017 there were 31,341 maternities in Wales. The median (range) number of maternities (including both midwifery- and obstetrician-led care) in the 12 hospitals was 2279 (538 to 5668). Throughout Wales in 2017, the vaginal delivery rate was 73.9% (9.9% assisted and 64.0% unassisted), 12.7% were elective caesarean sections, and 13.4% were non-elective caesarean sections [27].

**Fig. 1:**
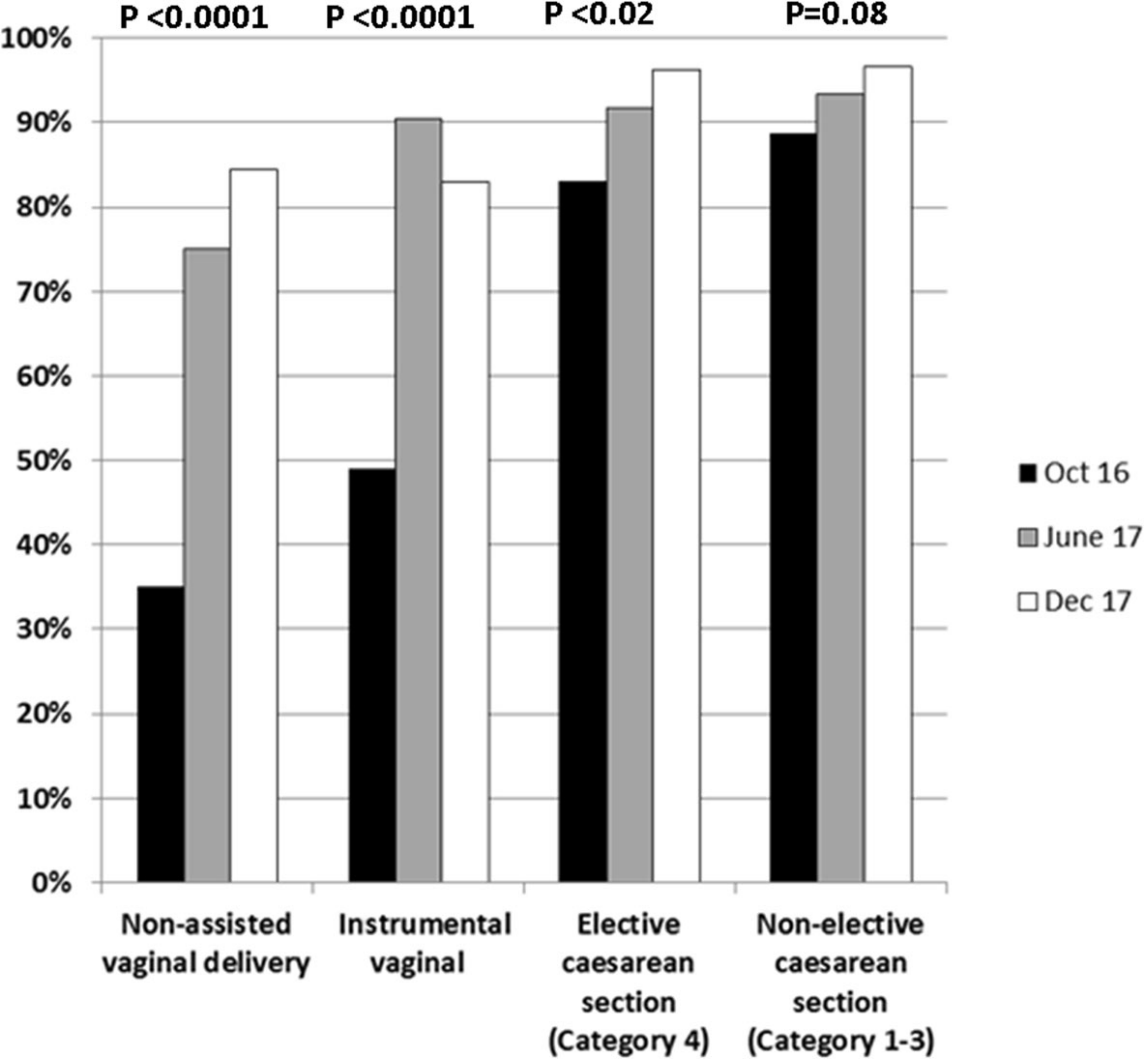
Objective measurement of all blood loss by mode of delivery. The proportion of maternities where all blood loss was objectively measured is shown for October 2016, before OBS Cymru started and subsequently in June and December 2017. The proportion of maternities where blood loss was measured was compared between October 2016 and December 2017 by the Chi Square test. Category 1 caesarean sections are where there is immediate threat to life of mother or fetus, category 2 maternal or fetal compromise that is not immediately life-threatening, category 3 is no maternal or fetal compromise but needs early delivery and category 4 is timed to suit woman and staff

The proportion of all maternities, by mode of delivery, that had QBL measurement performed prior to the OBS Cymru initiative and at two subsequent time points was assessed by audit and the results are shown in Fig. 1. QBL measurement increased from 52.1% in October 2016 (before the quality improvement initiative) to 87.8% in December 2017 (*P* < 0.001). An increase in the proportion of women having all blood loss measured was observed for all modes of delivery but was especially marked for vaginal deliveries where the proportion increased from 37.0 to 84.2%. The increase was statistically significant for assisted and unassisted vaginal deliveries and elective caesarean sections but not for non-elective caesarean sections, which had a high rate before the project (Fig. 1). The proportion of women who were included on the OBS Cymru database (with episodes of PPH > 1000 ml) who had blood loss measured quantitatively rather than by visually estimated increased from 525/677 (77.5%) in the first 3 months of 2017 to 714/718 (99.4%) in the last 3 months of 2017.

**Fig. 2:**
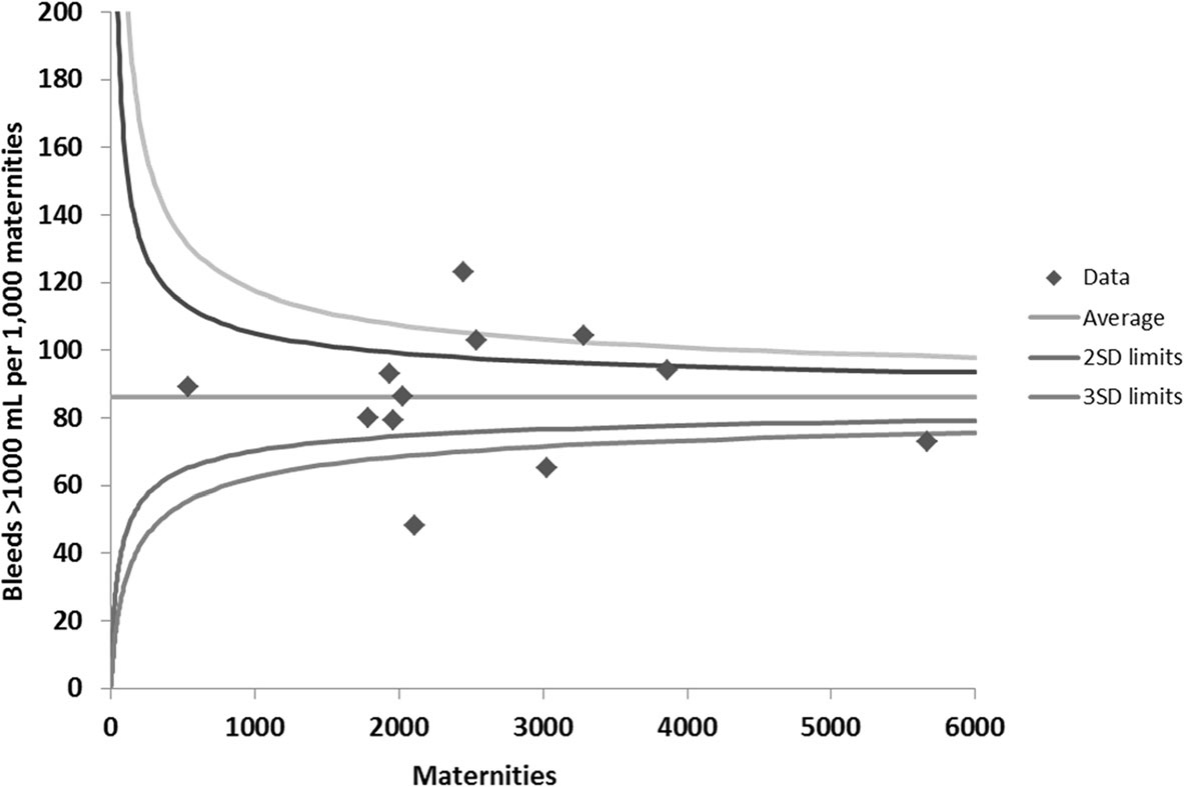
Incidence of blood loss more than 1000 mL at each maternity unit during 2017. Funnel plot of the number of bleeds more than 1000 mL per 1000 maternities during 2017 at each obstetric unit in Wales

In 2017 the incidence (95% CI) of cases with blood loss > 1000 mL in Wales was 8.6 (8.3 to 8.9) per 100 maternities whilst that of severe PPH (> 2000 mL) was 1.3 (1.2 to 1.4). The number of episodes of moderate and severe PPH, the incidence per 100 maternities and variation in hospitals providing maternity care across Wales is shown in Table 1 and Fig. 2. For comparison, the incidence of PPH between 1st April 2015 and 31st March 2016, before the introduction of quantitative measurement, was 5.1, 2.0 and 0.82 per 100 maternities for bleeds > 1000, > 1500 and > 2000 mL, respectively. Therefore the introduction of quantitative blood loss measurement in Wales was associated with was increased detection of PPH > 1000, > 1500 and > 2000 mL by 169, 165 and 159%, respectively.

**Table 1:** The incidence of moderate and severe postpartum haemorrhage in Wales in 2017

Volume of postpartum haemorrhage	Number of episodes of postpartum haemorrhage in Wales^a^	Incidence (95% CI) per 100 maternities in Wales^a^	Incidence of PPH per 100 maternities in the 12 maternity units^a^ in Wales Median (IQR)
**> 1000 mL**	2688	8.6 (8.3–8.9)	8.6 (7.8–9.6)
**> 1500 mL**	1039	3.3 (3.1–3.5)	3.6 (2.7–4.0)
**> 2000 mL**	404	1.3 (1.2–1.4)	1.5 (1.0–1.7)

The incidence of bleeds > 2000 mL and > 1500 are subgroups of > 1000 mL. There were 1649 bleed between > 1000 and 1500 mL and 635 between > 1500 and 2000 mL. ^a^Data include midwifery and obstetrician-led care from all 12 maternity units in Wales. The incidence (95% CI) is for all maternities in Wales with the denominator *n* = 31,341. The incidence at each of the 12 obstetric units is described by median and interquartile range (IQR) to describe the variation between hospitals

The incidence of PPH, dependent on mode of delivery, is shown in Table 2. PPH of all severities was most common for non-elective caesarean sections followed by instrumental vaginal deliveries and least common for unassisted vaginal deliveries.

**Table 2:** Incidence of postpartum haemorrhage in Wales dependent on mode of delivery

	Unassisted vaginal	Instrumental vaginal	Elective caesarean section	Non-elective caesarean section
**Estimated maternities in Wales in 2017**	20,055	3106	4193	3987
**Number (95% CI) blood loss > 1000 mL per 100 maternities**	4.9 [4.6–5.2]	18.4 [17.1–19.8]	8.5 [7.7–9.4]	19.8 [18.6–21.0]
**Number (95% CI) blood loss > 1500 mL per 100 maternities**	2.1 [1.9–2.3]	6.8 [6.0–7.78]	2.9 [2.4–3.4]	8.2 [7.4–9.1]
**Number (95% CI) blood loss > 2000 mL per 100 maternities**	0.80 [0.68–0.93]	2.7 [2.2–3.3]	1.0 [0.74–1.4]	3.3 [2.8–3.9]

The OBS Cymru database recorded information about blood loss > 1000 mL and so could not be used to investigate the incidence of PPH between 500 and 1000 mL. Data about blood loss at the time of all births, included both midwifery- and obstetrician-led care, linked to mode of delivery, were available from four maternity units. The incidence of PPH ≥500 mL for all maternities in Wales in 2017 was 34.0%, according to the NHS Wales Informatics Service. The incidence (95% CI) of PPH ≥500 mL in the four maternity units where more detailed information was available was 33 (32–34) per 100 maternities demonstrating that these centres were representative of the country as a whole. The median total blood loss for all births was similar in each of the four maternity units for all modes of delivery, irrespective of the size of the unit and case mix. The median (IQR) blood loss for all deliveries at the four centres combined was 350 (219–600) mL. Unassisted vaginal deliveries had the lowest blood loss, median 300 mL, but instrumental vaginal deliveries (median 500 mL) were similar to caesarean sections. The incidence of bleeds ≥500 mL in the 4 units varied between 30.9 and 40.7% (Table 3). There was remarkable consistency between maternity units for unassisted vaginal deliveries with an overall incidence of 18.2% (range 17.4 to 19.1%). Wider variation in the incidence of PPH ≥500 mL was observed between centres for non-elective caesarean sections.

**Table 3:** Total blood loss for all maternities and incidence of bleeds > 500 mL

		Total measured blood loss (mL) Median (25th to 75th percent)					Postpartum haemorrhage ≥ 500 mL Incidence (95% CI)/100 maternities				
Unit	Maternities included in analysis	All maternities	Unassisted vaginal delivery	Instrumental vaginal delivery	Elective caesarean section Category 4	Non Elective caesarean section Category 1–3	All maternities	Unassisted vaginal delivery	Instrumental vaginal delivery	Elective caesarean section Category 4	Non Elective caesarean section Category 1–3
1	6622	300 (200–500)	300 (200–400)	500 (300–800)	450 (300–700)	500 (350–750)	30.9 [29.8–32.0]	18.1 [16.9–19.3]	53.8 [50.6–57.0]	48.8 [45.4–52.3]	55.4 [51.8–59.0]
2	241	421 (265–651)	301 (199–450)	532 (344–961)	800 (533–1165)	602 (438–890)	40.7 [34.7–47.0]	17.4 [12.0–24.6]	53.8 [35.5–71.2]	88.6 [74.0–95.5]	69.0 [54.0–80.9]
3	925	400 (250–732)	283 (157–448)	600 (400–1000)	559 (361–820)	700 (437–940)	40.4 [37.3–43.6]	18.6 [15.4–22.3]	66.7 [56.1–75.8]	55.0 [47.5–62.2]	70.7 [63.9–76.7]
4	892	400 (245–700)	300 (199–450)	500 (333–860)	543 (413–850)	677 (454–911)	39.1 [36.0–42.4]	19.1 [16.0–22.7]	55.7 [45.3–65.6]	68.3 [59.6–76.0]	71.7 [64.4–78.0]
All	8680	350 (219–600)	300 (200–400)	500 (300–850)	500 (300–750)	550 (369–850)	33.0 [32.0–34.0]	18.2 [17.2–19.3]	54.9 [52.0–57.8]	53.0 [50.1–55.9]	60.9 [58.0–63.7]

## Discussion

In this manuscript we report on the national incidence of PPH after standardised, QBL measurement was adopted by all 12 hospitals providing maternity care in Wales. The method identified an incidence of PPH > 1000 mL of 8.6% and > 2000 mL of 1.3% which was an increase of about 160% compared to before the initiative. In a subgroup of four maternity units of widely varying size, the incidence of PPH ≥500 mL was between 30.9 and 40.7%. The incidence of PPH reported here, after the introduction of QBL measurement, is higher than in many previous reports and has potential implications for clinical practice and research.

The initiative led to an increase in QBL measurement for all modes of delivery. Improvement was observed in the theatre environment even though high compliance with quantitative measurement had been present before the programme. This demonstrates that quantitative measurement of blood loss is feasible in all hospital-based maternity settings, including midwifery- and obstetrician-led care, and outside of routine hours. The increased proportion of women with measured blood loss has been sustained over at least 1 year and been embedded in undergraduate midwifery education in Wales.

A systematic review between 1997 and 2002 reported an incidence of PPH ≥500 mL of 7.2% for subjectively and 10.4% for quantitative assessed blood loss [4]. A study from France reported PPH ≥500 mL at 10% [5] for quantitative measured blood loss and an international systematic review reported 14.2% [3]. These figures are at least 50% lower than the incidence of 30.9–40.7% observed in Wales in 2017 where quantitative measurement was started at delivery and the same method, confirmed by audit, was used throughout the country. In the context of clinical studies performed more recently, a UK group reported the incidence of PPH ≥500 mL to be 33.7% [11] and in Australia 22% [6] which are in line with our findings established in routine care.

Systematic reviews report an incidence of PPH > 1000 mL for quantitatively measured blood loss to be between 2% [5] and 4.2% [3]. A Swedish study reported bleeds > 1000 mL to be 4.7% [28]. These results are again about 50% lower than the 8.6% reported here. A recent interventional clinical trial of women having a vaginal delivery that measured blood loss with under buttock drapes, reported PPH > 1000 mL to be 8.1% with intramuscular oxytocin [29] and 4.6% with intravenous oxytocin, similar to our results in one arm of the study.

It is very likely that quantitative measurement of blood loss from delivery with standardisation across hospitals providing maternity care contributed to the high incidence of PPH observed in this report. Shields et al. reported a quality improvement programme in California that introduced blood loss measurement as part of a comprehensive approach to PPH and found that bleeds > 1500 mL increased from 2.7 to 4.3% (a 159% increase) [9]. This is similar to the incidence reported in Wales where we observed an increase of 165% from 2 to 3.3%. The observed increase in PPH > 1500 mL is remarkably similar between the two studies and indicates that quantitative measurement detects more cases of severe PPH than visual estimation. Whilst the possibility that there was an actual 165% increase in PPH over a 1 year period in Wales cannot be excluded, this is unlikely because the change is so large and the demographics of the population and obstetric practice did not change and the increase is almost identical to that observed by Shields et al. During this time prophylactic uterotonics were recommended for active management of the 3rd stage of labour for all deliveries in Wales [30]. The different methods of data collection between 2016 and 2017 may have also contributed to the observed increase. In OBS Cymru, maternity units measure all bleeding, rather than start at the time of clinical concern [1], and do this cumulatively in real time. This is likely to increase identification of cases where blood loss is between 500 and 1500 mL. Cumulative measurement alerts clinicians to progression of bleeding and prompts escalation of care to more senior clinicians at an appropriate time, as described in guidelines and previous studies [1, 8].

We observed a large difference in the incidence of PPH between different modes of delivery with instrumental vaginal deliveries and emergency caesarean sections being substantially higher than elective caesarean section and unassisted vaginal delivery. This may, in part, be due to difference in the modes of volumetric and gravimetric measurement. However, the correlation between fall in haemoglobin for each mode of delivery was similar (manuscript accepted), indicating that the method worked adequately well in both situations. This suggests that the difference in observed blood loss is unlikely to be due to the method of measurement alone. Stafford et al. reported that if visual estimation is used blood loss appears to be similar between spontaneous and operative vaginal deliveries (median 250 vs 300 mL) but dissimilar if blood loss is calculated based on fall in haemoglobin (574 mL vs 728 mL) [31].

It is reported that blood loss measurement alone does not reduce PPH rates [6, 14]. A cluster randomised study comparing systematic use of a collector bag with visual estimation found no difference between the two arms [32]. A Cochrane review identified two studies that met the pre-specified inclusion criteria and concluded that there were insufficient data to support the use of either estimated or quantitative blood loss, highlighting the need for high quality trials in the field [21]. Cumulative, quantitatively measured blood loss has potential value when integrated into a multidisciplinary escalation policy to inform team interventions with the aim of preventing small or moderate bleeds becoming severe. Improved outcomes have been shown in the context of this integrated approach both in single centre reports and large quality improvement programmes [9, 23].

The main strength of this report is that it is a large, prospective, real world, consecutive cohort of all maternities in Wales covering both midwifery- and obstetrician-led births. It provides new and consistent data on blood loss after different modes of birth. The report involves multiple maternity units of different sizes, case mix and staffing levels. The rates of vaginal and caesarean delivery across Wales are representative of the UK suggesting that they are applicable to other regions (https://files.digital.nhs.uk/pdf/l/1/hosp-epis-stat-mat-repo-2016-17.pdf, accessed 14th Mar 2020). There was a high level of sustained compliance with quantitatively measured blood loss and remarkably consistent results.

Limitations of the analysis are that blood loss data linked to mode of delivery for all maternities are available from only 4 units, although this subgroup includes more than 8000 maternities and the rate of PPH ≥500 mL was almost identical to the whole of Wales. Quantitative measurement was performed at all sites although we cannot be certain of the accuracy of measurement. It is likely, however, that the findings are more accurate than for visually estimation. Accurate quantification of amniotic fluid remains a challenge for both quantitative and estimated techniques. In addition, QBL measurement was not integrated into community based midwifery led births until 2019. During 2017, all women giving birth in community-based midwifery-led birth facilities who experienced PPH > 1000 ml were transferred to the closest hospital and included in the receiving institutions data collection.

In conclusion, quantitative measurement of peri-partum blood loss has become standard practice in Wales and has been associated with improved detection of PPH. Standardisation of blood loss measurement is important for understanding trends in the incidence of PPH and how bleed volume affects PPH-related morbidity. Defining the amount of blood loss to be expected at the time of delivery is important for assessing the impact of interventions aiming to reduce PPH and to compare research studies. Although uncontrolled reports in the context of quality improvement suggest that cumulative, quantitative measurement of blood loss, integrated into co-ordinated multidisciplinary care of PPH, improves outcomes such as need for transfusion [9] these findings need to be investigated in high quality trials to assess whether clinically relevant outcomes are genuinely improved.

## Data Availability

The datasets used and/or analysed during the current study are available from the corresponding author on reasonable request.

## Abbreviations

PPH: Postpartum haemorrhage
QBL: Quantitative blood loss
RCOG: Royal College of Obstetrics and Gynaecology
CI: Confidence intervals
